# Use of pulsatile gonadotropin-releasing hormone (GnRH) in patients with functional hypothalamic amenorrhea (FHA) results in monofollicular ovulation and high cumulative live birth rates: a 25-year cohort

**DOI:** 10.1007/s10815-022-02656-0

**Published:** 2022-11-15

**Authors:** Philipp Quaas, Alexander M. Quaas, Manuel Fischer, Christian De Geyter

**Affiliations:** 1 Department of Obstetrics and Gynecology, University Hospital, University of Basel, Spitalstrasse 21, CH-4056 Basel, Switzerland; 2 Reproductive Medicine and Gynecological Endocrinology (RME), University Hospital, University of Basel, Vogesenstrasse 134, CH-4031 Basel, Switzerland; 3 University of Basel, Petersplatz 1, CH-4001 Basel, Switzerland

**Keywords:** Functional hypothalamic amenorrhea (FHA), Hypothalamic hypopituitarism, Gonadotropin-releasing hormone (GnRH), Pulsatile GnRH pump, Ovulation induction (OI)

## Abstract

**Purpose:**

To analyze outcomes of pulsatile administration of gonadotropin-releasing hormone (GnRH) in infertile women diagnosed with functional hypothalamic amenorrhea (FHA).

**Methods:**

A single-center retrospective cohort study was conducted from 1996 to 2020. Sixty-six patients with the diagnosis FHA that underwent therapy using the pulsatile GnRH pump for conception were included and analyzed. The primary outcome was the live birth rate (LBR). Secondary outcomes were the number of dominant follicles, ovulation rate, biochemical pregnancy rate (BPR), clinical pregnancy rate (CPR), miscarriage rate, and multiple pregnancy rate. A matched control group was selected to compare the birth weight of newborn children.

**Results:**

During the study period, 66 patients with FHA underwent 82 treatments (14 of 66 patients had more than one treatment) and a total of 212 cycles (ovulation induction attempts) using pulsatile GnRH. The LBR per treatment was 65.9%. The ovulation rate per cycle was 96%, and monofollicular ovulation was observed in 75% of cycles. The BPR per treatment was 80.5%, and the cumulative CPR per treatment was 74.4%. The miscarriage rate was 11.5%. One dizygotic twin pregnancy was observed (1.6%). Average newborn birth weight (NBW) from patients with FHA was comparable to the control group.

**Conclusion(s):**

In patients with FHA, excellent pregnancy rates were achieved using the subcutaneous GnRH pump. The high cumulative LBR with normal NBW as well as low rates of multiple gestation indicate that the pulsatile GnRH pump represents a safer and more physiologic alternative to ovulation induction with injectable gonadotropins.

**Trial registration:**

Ethics Committee Northwest and Central Switzerland (Ethikkommission Nordwest- und Zentralschweiz - EKNZ) - Project-ID 2020-01612.

## Introduction

Functional hypothalamic amenorrhea (FHA) is characterized by dysfunction of the hypothalamic-pituitary-ovarian (HPO) axis, resulting in chronic anovulation and secondary amenorrhea as well as infertility due to low circulating gonadotropin levels. Up to 5% of adult women are affected by FHA, also termed World Health Organization (WHO) group I hypogonadotropic hypogonadal anovulation. Known causes include high levels of stress and exercise, genetic predisposition, and psychiatric disorders such as anorexia nervosa (AN) [1–6]. There is evidence that patients with a lower body mass index (BMI) are more likely to deliver children with a significantly lower birth weight [7–10].

The “critical weight hypothesis” postulates that a certain threshold of body mass or body fat is necessary to establish regular cycles [11, 12]. The absence of ovulation resulting in amenorrhea is potentially harmful to patients with regard to their cardiovascular and bone health. The term “female athlete triad” refers to the clinical constellation of amenorrhea, eating disorders, and osteopenia or osteoporosis [1, 3, 13]. The ideal strategy for the treatment of anovulatory infertility in this patient population is to restore regular ovulatory cycles through an adaptation of lifestyle. Reducing chronic stress and/or high-intensity exercise as well as normalization of BMI can reactivate the HPO-axis and thus reinstate regular menstrual cycles. In many cases, lifestyle changes are hard to achieve and may not lead to the resumption of regular menstrual cycles [12, 14]. If medical intervention is needed, hormonal replacement therapy is indicated to prevent premature bone loss.

Elucidation of the molecular structure of gonadotropin-releasing hormone (GnRH) by Schally and co-workers in 1971 [15] and the discovery of the pulsatile nature of GnRH secretion by Knobil and co-workers in 1981 [16–19] laid the physiologic foundation for the development of the pulsatile GnRH pump for patients with hypothalamic anovulation. Leyendecker and Wildt developed the first version of the pump (Zyklomat Pulse®; Ferring Holding S.A., Saint-Prex, Switzerland) in 1982 [20, 21] as a promising novel therapeutic option to restore regular ovulation in patients with FHA wishing to conceive [21, 22].

Although the pulsatile administration of GnRH has been approved by the Food and Drug Administration (FDA), injectable gonadotropins are predominantly used for ovulation induction (OI) in patients with FHA. A recent systematic review and meta-analysis concluded that the subcutaneous pulsatile administration of GnRH is superior in terms of effectiveness and safety and should be the preferable method of ovarian stimulation in this patient collective [23].

The currently available subcutaneous GnRH pump (Lutrepulse®; Ferring Holding S.A., Saint-Prex, Switzerland) consists of a “manager” and a “pod”. The manager is programmed by the user (doctor or patient) and controls the pod to release a certain dose of GnRH (Lutrelef®) subcutaneously every 90 min.

Prior studies have demonstrated the safety and efficacy of the GnRH pump in patients with FHA [24–26] as an alternative to injectable gonadotropins, which are associated with risks of multifollicular ovulation, potentially resulting in multiple pregnancy and ovarian hyperstimulation syndrome (OHSS) [23].

This study aimed to retrospectively analyze the long-standing experience of treatment of patients with FHA using the GnRH pump in a Swiss University–based tertiary referral center over the last 25 years and to compare neonatal outcomes with a matched control group of ovulatory women with infertility during the same time period.

## Materials and methods

### Study design

We performed a retrospective single-center cohort study of all patients with FHA undergoing OI using the GnRH pump between 1996 and 2020 and compared the outcomes with those of a matched control group consisting of ovulatory women with comparable ages and achieving pregnancy naturally during the same time period.

### Inclusion criteria

**Fig. 1:**
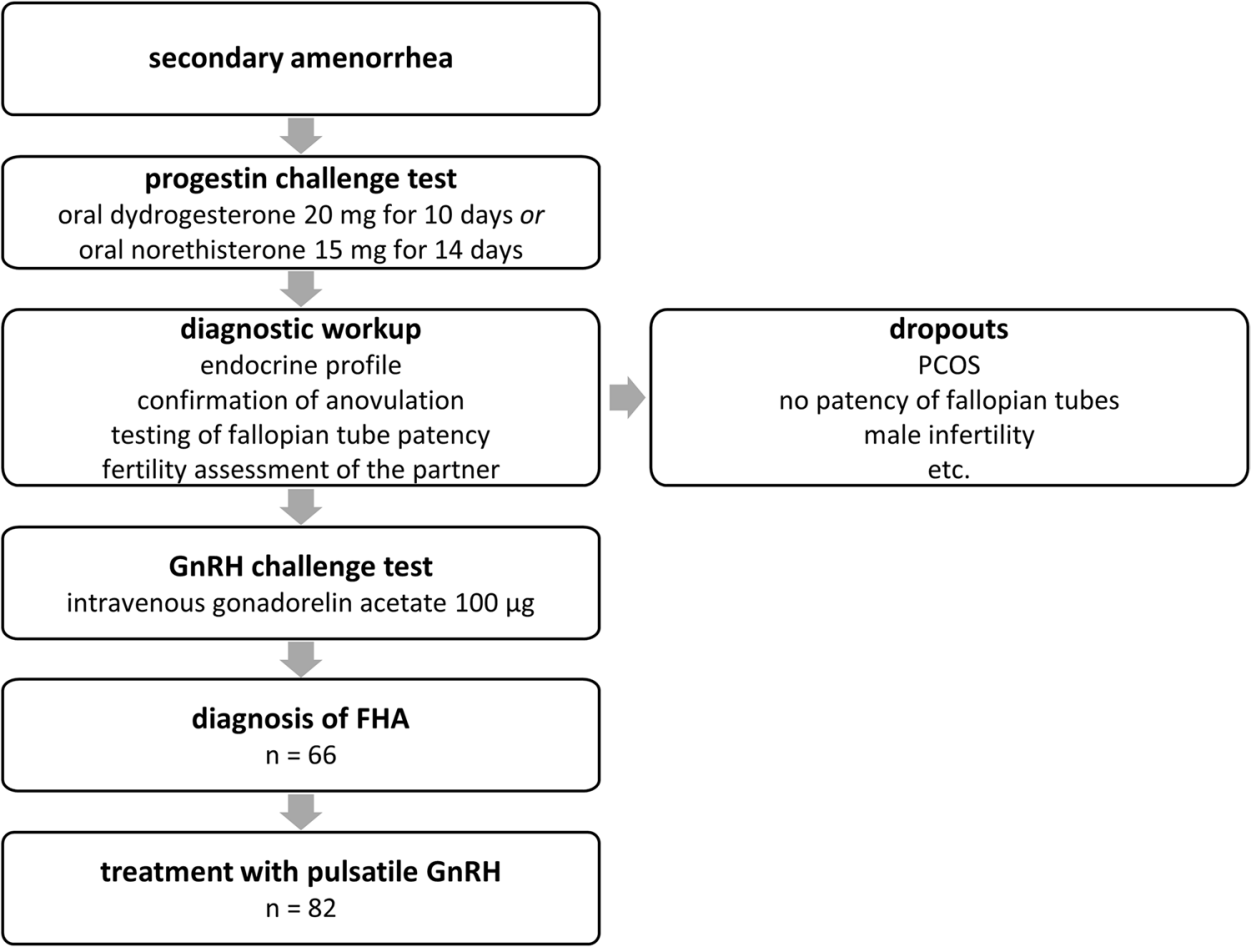
Study flow chart. mg = milligrams, µg = micrograms, PCOS = polycystic ovary syndrome, GnRH = gonadotropin-releasing hormone, FHA = functional hypothalamic amenorrhea

Inclusion criteria for the study group were age between 20 and 40 years as well as a diagnosis of FHA. FHA was diagnosed based on factors such as low circulating follicle-stimulating hormone (FSH), luteinizing hormone (LH), and estradiol levels as well as an inadequate response of FSH and LH to an intravenous bolus of GnRH. Figure 1 illustrates the diagnostic algorithm from patient presentation with secondary amenorrhea to treatment with pulsatile GnRH in order to conceive. Inclusion criteria for the matched control group were a diagnosis of unexplained infertility and natural conception during the diagnostic workup.

### Exclusion criteria

Patients with diagnoses such as polycystic ovarian syndrome (PCOS), hyperprolactinemia, Kallman syndrome, and acute AN were excluded. Infertile couples with missing patency of the fallopian tubes or abnormal semen quality (as diagnosed with conventional semen analysis according to the WHO criteria) were also excluded from treatment with pulsatile administration of GnRH.

### Diagnostic and therapeutic protocols

Over the study period, the standard methodology for the diagnostic workup and treatment with pulsatile GnRH remained unchanged (Fig. 1). After recording of medical history and performing a physical examination, patients with secondary amenorrhea were subjected to a progestin challenge test followed by an endocrine profile (with FSH, estradiol, LH, prolactin, androgens) and serial vaginal ultrasound monitoring to confirm anovulation. The patency of the fallopian tubes was tested and the fertility of the partner assessed. All patients then received a GnRH (challenge) test following standard protocols. Once a diagnosis of FHA was established, treatment with pulsatile administration of GnRH was initiated.

From 1996 to 2010, patients were treated with the previous version of the pulsatile GnRH pump (Zyklomat Pulse®; Ferring Holding S.A., Saint-Prex, Switzerland).

Starting in 2011, the currently available pulsatile GnRH pump was used for treatment (Lutrepulse®; Ferring Holding S.A., Saint-Prex, Switzerland).

The latter device consists of a pod and a drug delivery manager (DDM). The DDM controls the pod to administer 10 µg of subcutaneous GnRH (Lutrelef®, Ferring Holding S.A., Saint-Prex, Switzerland) every 90 min. The pod was placed on different cutaneous locations, usually on the lower abdomen and was changed every third day. Activities of daily life, including swimming, were still possible during ongoing treatments. Once therapy with the GnRH pump was initiated, patients had regular transvaginal ultrasounds to monitor follicular growth. Upon development of at least one dominant follicle (follicle size > 14 mm), patients were advised to have intercourse. After that, ovulation was confirmed via ultrasound of a corpus luteum, and an human chorionic gonadotropin (hCG) level checked at least 9 days post ovulation.

### Primary and secondary outcomes

The primary outcome was live birth rate (LBR) per treatment. Secondary outcomes included the number of dominant follicles (size > 14 mm) and ovulation rate per cycle (data available for 2014–2020), biochemical pregnancy rate (BPR), clinical pregnancy rate (CPR), miscarriage rate, and multiple pregnancy rate. Baseline characteristics including age, BMI (kg/m^2^), smoking status, anti-Müllerian hormone (AMH), antral follicle count (AFC), endocrine profiles, and lifestyle parameters including exercise were recorded.

### Control group

Among the 66 patients treated with pulsatile administration of GnRH, data on newborn birth weight (NBW) was available in 45 women. In order to evaluate the effect of FHA on the condition of the newborn, matched controls of ovulatory women not diagnosed with FHA were retrieved from the local database (FertiMed®, Reinach, Switzerland). Matching was performed based on patient age and year of conception. The NBW from patients with FHA that conceived using the pulsatile GnRH pump was compared with the one from matched controls.

### Use of the GnRH pump (Lutrepulse®)

The pump consisting of a pod and a DDM was used and programmed according to the guidelines of the manufacturer (Ferring Holding S.A., Saint-Prex, Switzerland). Maintenance, management, and programming of the device were performed by healthcare professionals in this study.

### Statistical analysis

Statistical analysis was done with the Mann–Whitney *U* test and the chi-square test to analyze non-parametric data. A *p* value of < 0.05 was considered statistically significant.

### Institutional review board (IRB) approval

IRB approval was obtained by the local ethics committee (Ethics Committee Northwest and Central Switzerland—Project-ID 2020–01612).

### Funding and support disclosure

The corresponding author has no official relationship to or with the manufacturer of Lutrepulse® and Lutrelef® (Ferring Holding S.A., Saint-Prex, Switzerland). AMQ and CDG act as consultants and/or occasional speakers for the company. All authors declare that there was no industry support of any kind that had been received in order to conduct this study.

## Results

During the study period, 66 patients underwent 82 separate treatments (12 patients had 2 treatments, 2 patients had 3 treatments) for a total number of 212 cycles (ovulation induction attempts using the GnRH pump). During one treatment episode, consecutive ovulation induction cycles were undertaken. The patient baseline characteristics as well as GnRH challenge test results are shown in Table 1. Mean age of the patients was 31.6 years, and mean BMI was 20.2 kg/m^2^. Mean AMH was 35.2 pmol/l (equivalent to 4.9 ng/ml). The majority of patients consisted of non-smokers. The ovulation rate per cycle was 96% and monofollicular ovulation (one follicle of at least 14 mm or more at the time of ovulation) was observed in 75% of cycles (data available for 2014–2020). The cumulative CPR per treatment was 61 out of 82 (74.4%), and the overall LBR per treatment was 54 out of 82 (65.9%). The miscarriage rate was 7 out of 61 (11.5%). There was one dizygotic twin pregnancy for a multiple pregnancy rate of 1 out of 61 (1.6%) clinical pregnancies or 1 out of 54 (1.9%) live births, respectively (Table 2).

**Table 1:** Characteristics of study cohort, patient baseline characteristics and results from routinely performed gonadotropin-releasing hormone (GnRH) challenge test

a) Characteristics of study cohort	*n*	SD
Overall patients with FHA	66	
Overall treatments with GnRH pump	82	
Overall cycles with GnRH pump	212	
Menstrual cycles per treatment	2.59	1.68
Menstrual cycles per treatment if clinical pregnancy was achieved	2.11	1.08
b) Patient baseline characteristics	Mean	SD
Age [y]	31.6	3.7
BMI [kg/m^2^]	20.2	4.0
AMH [pmol/l] (since 2007)	35.2	21.0
AFC [n]	23.9	12.2
Baseline FSH [IU/l]	5.7	2.7
Baseline LH [IU/l]	3.8	3.0
Estradiol [pmol/l]	89.1	105.1
Testosterone, total [nmol/l]	0.6	0.4
SHBG [nmol/l]	71.9	33.3
DHEAS [umol/l]	3.6	1.7
Prolactin [mU/l]	236.2	179.0
c) GnRH challenge test	Mean	SD
FSH 0’ [IU/l]	5.65	1.92
FSH 25’ [IU/l]	10.68	3.85
FSH 45’ [IU/l]	12.12	4.45
LH 0’ [IU/l]	3.98	2.99
LH 25’ [IU/l]	25.21	18.01
LH 45’ [IU/l]	25.30	19.01

*n* number, *SD* standard deviation, *FHA* functional hypothalamic amenorrhea, *GnRH* gonadotropin-releasing hormone, *BMI* body mass index, *AMH* anti-Müllerian-hormone, *AFC* antral follicle count, *FSH* follicle-stimulating hormone, *LH* luteinizing hormone, *SHBG* sex hormone-binding globulin, *DHEAS* = dehydroepiandrosterone sulfate

**Table 2:** Primary and secondary outcomes: overview of primary and secondary outcomes of the study

Outcome	Outcome (n/total)	%
BPR per cycle	66/212	31.1
CPR per cycle	61/212	28.8
LBR per cycle*	54/212*	25.5
Multiple pregnancy rate	1/61	1,6
Miscarriage rate (biochemical)	5/66	7.6
Miscarriage rate (clinical)	7/61	11.5
Congenital malformation	0/54	0
Twin births	1/54*	1.9
BPR per treatment	66/82	80.5
CPR per treatment	61/82	74.4
LBR per treatment	54/82*	65.9
*Twin birth was calculated for dataset as one live birth		

*n* number, *BPR* biochemical pregnancy rate, *CPR* clinical pregnancy rate, *LBR* live birth rate

The mean NBW in the FHA cohort was not statistically different from the NBW of the matched control group of infertile patients with no history of FHA and ovulatory cycles (Table 3).

**Table 3:** Comparison of newborn birth weight (NBW) after therapy with pulsatile gonadotropin-releasing hormone (GnRH) in patients with functional hypothalamic amenorrhea (FHA) and after natural conception in subfertile ovulatory patients

	FHA (WHO I)		Matched controls		*p* value
Numbers (*n*)	45		45		
Age (y)	31.1	0.6	31.6	0.6	0.509
BMI (kg/m^2^)	19.5	0.4	22.8	0.7	< 0.00001
AMH (pmol/l)	33.9	3.2	32.6	5.4	0.441
AFC (n)	24.0	2.0	21.1	2.3	0.112
FSH (IU/l)	5.4	0.4	7.6	0.7	0.001
LH (IU/l)	6.6	0.4	7.3	0.7	< 0.00001
*NBW (g)*	*3188*	*68*	*3265*	*65*	*0.465*

Median values are given together with the standard error of the mean (SEM). *FHA* functional hypothalamic amenorrhea, *WHO* World Health Organization, *BMI* body mass index, *AMH* anti-Müllerian hormone, *AFC* antral follicle count, *FSH* follicle-stimulating hormone, *LH* luteinizing hormone, *NBW* newborn birth weight

No significant adverse effects were reported by any of the patients in the study.

## Discussion

In this study, further evidence is provided that the subcutaneous pulsatile administration of GnRH is the preferable method of ovulation induction in women with FHA-related infertility. In most patients, monofollicular ovulation was achieved in the first treatment cycle with the GnRH pump. Live birth rates were high and the multiple birth rate was comparable to that of the general population.

**Fig. 2:**
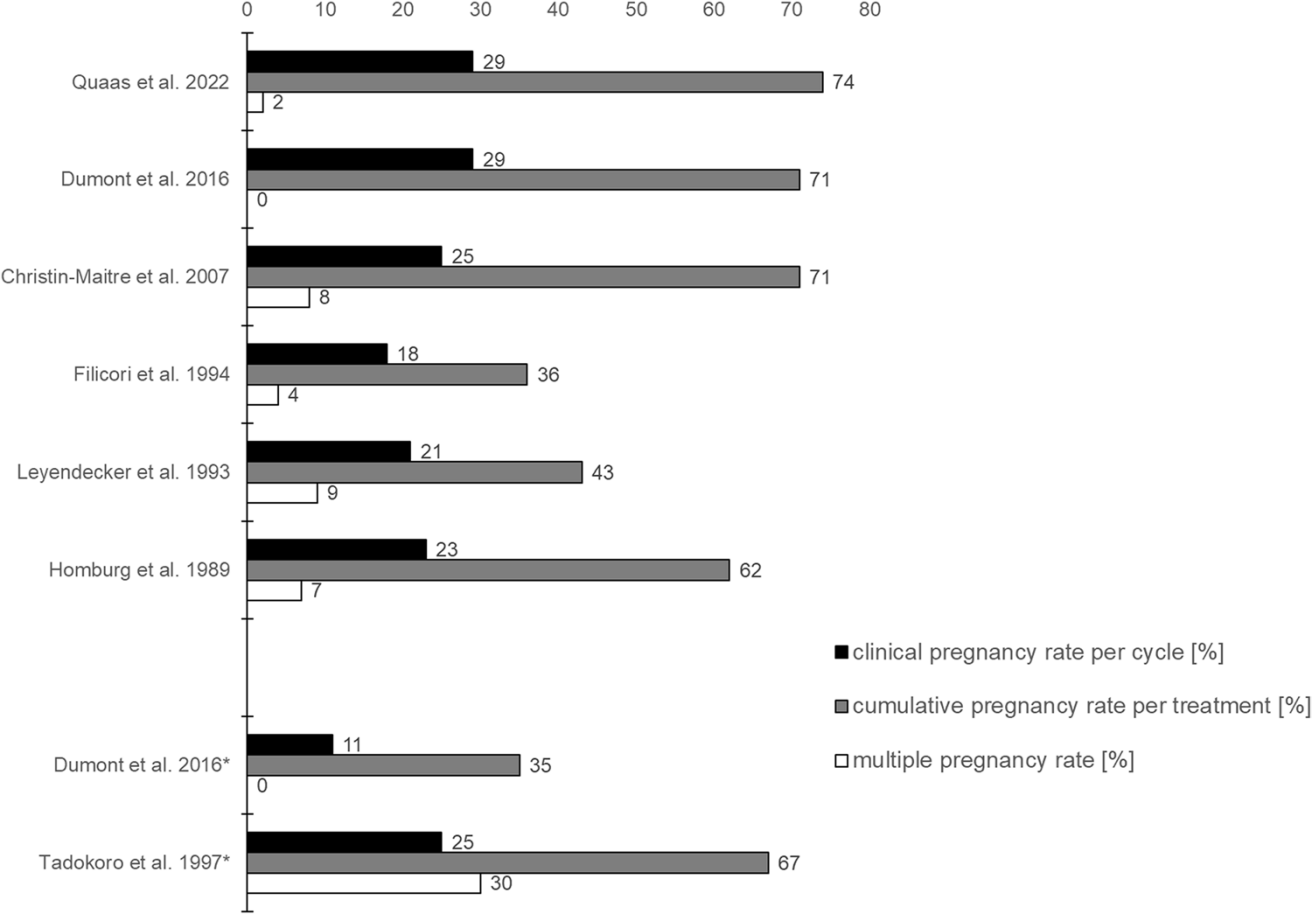
Pregnancy outcomes of the five largest cohorts of functional hypothalamic amenorrhea (FHA) patients treated with pulsatile gonadotropin-releasing hormone (GnRH), along with the outcomes of our study cohort. Dumont et al. 2016* and Tadokoro et al. 1997* demonstrate outcomes of FHA patients treated with injectable gonadotropins in comparison

The superiority of pulsatile GnRH administration compared to injectable gonadotropins in patients with FHA is supported by previous literature [27]: Ovulation rates were 96% with GnRH and 84% with gonadotropins (*p* < 0.05), and monofollicular ovulatory cycles were more prevalent when using the GnRH pump (75% vs 53%, *p* < 0.05). Treatment using injectable gonadotropins is associated with a higher number of treatment cycles as well as higher and more variable doses of hormones in order to achieve pregnancies [27]. Treatment using pulsatile GnRH demonstrates equal or superior outcomes compared to injectable gonadotropins [28] without gonadotropin treatment-related complications of multiple pregnancies and miscarriages [29] (Fig. 2).

Although there are reports that show significantly lower NBW from patients with a lower BMI [7–10], children born from patients with FHA in this study cohort (mean BMI 20.2 kg/m^2^) had a birth weight comparable to children born from ovulatory patients (Table 3). Further studies are required to confirm this observation. There were no severe adverse events reported by any of the subjects in the study.

While patients with active psychiatric disorders such as acute AN were excluded from pulsatile GnRH treatment for conception, the retrospectively analyzed study cohort included women with a history of recovered AN. Despite recovery from AN and weight gain to normal BMI the menstrual cycle does not resume in a considerable number of cases. Recent investigations have demonstrated alterations in inflammatory markers [30–32] as well as the gut microbiome and microbial metabolites [33, 34] in this patient collective. Research in these areas may help uncover additional therapeutic options for patients with recovered AN in order to reinstate ovulatory cycles and conceive.

The cohort of FHA patients in this study represents one the largest of its kind treated with the GnRH pump [23]. Figure 2 compares the outcomes with other studies where patients have been treated using pulsatile GnRH [22, 24, 28, 35, 36] or injectable gonadotropins [28, 29]. A large number of older studies was flawed by the inclusion of patients with PCOS along with FHA patients. This may have contributed to the higher rate of spontaneous abortions [37] and highlights the importance of a thorough diagnostic workup prior to initiation of pulsatile GnRH treatment (Fig. 1). Furthermore, the higher pregnancy rates reported in this study may also be a result of cumulative pharmacological and technological advances over the last decades.

The findings presented in this study support the use of the pulsatile GnRH pump as the preferable option in patients with FHA wishing to conceive, as it is associated with a high cumulative live birth rate along with a low multiple pregnancy rate.

While older versions of the GnRH pump were bulky and cumbersome, newer devices are smaller and more practical. The new portable pump system, which consists of a pod and a DDM, can be used and programmed by the healthcare professional as well as the patient herself, reducing the number of office visits. While pod detachment or dislocation may rarely be observed with the use of the GnRH pump, no actual adverse events, such as infection or bleeding, occurred. Patients were not impaired in their daily activities during therapy with the device.

Use of the GnRH pump is associated with significant costs of material, medication, and monitoring. It also requires the learning of technical skills by providers and patients. Despite these potential pitfalls, our data support a more widespread use of the GnRH pump, including in the United States of America (USA) where it is FDA-approved but not commercially available. It is feasible that this may change as new evidence becomes available: a USA-based multicenter, double-blind, randomized, placebo-controlled trial evaluating three different doses of pulsatile GnRH administered via a subcutaneous pump for ovulation induction in patients with FHA was completed recently (ClinicalTrials.gov Identifier: NCT01976728). The use of pulsatile administration of GnRH has also been explored for other indications, such as in patients with Down syndrome (DS) to improve cognitive function [38].

## Conclusion

In patients with FHA, monofollicular ovulation can be achieved when using a portable pump delivering GnRH in a pulsatile fashion. Pregnancy outcomes, including multiple pregnancy rates, are similar to those observed in natural conception. The portable pulsatile GnRH pump represents a safe, reliable, and physiologic approach for OI.

## Data Availability

The authors confirm that the data supporting the findings of this study are available within the article [and/or] its supplementary materials. The data that support the findings of this study are available from the corresponding author, Philipp Quaas, upon reasonable request.

## Data sharing

The authors declare that all data will be shared and made available upon request after publication in accordance with the ICMJE data sharing guidelines.
